# Changes in Medicare Reimbursement for Top Burn Surgery Procedures by State Between 2011 and 2022

**DOI:** 10.7759/cureus.69464

**Published:** 2024-09-15

**Authors:** Jack G Allen, Alexander Dorius MBA, Carson Bateman, Colton Shepherd, John Griswold, Alan Pang

**Affiliations:** 1 Department of Surgery, Texas Tech University Health Sciences Center, Lubbock, USA; 2 Department of Surgery, University of Utah School of Medicine, Salt Lake City, USA

**Keywords:** burn surgery, geographic variation, healthcare insurance, interstate variation, medicare reimbursement

## Abstract

This study analyzed the geographic variation in annual Medicare reimbursement changes for common burn surgery procedures from 2011 to 2022 to clarify trends in reimbursement. The Center for Medicare and Medicaid Services’ Physician fee schedule database was analyzed to find state-by-state reimbursement rates for the most common burn surgery procedures. Physician reimbursement was adjusted for inflation utilizing the consumer price index. Procedures were weighted according to frequency, and an inflation-adjusted percent change was identified for each state. Since 2011, the inflation-adjusted Medicare reimbursement for the top burn surgery procedures for all U.S. states decreased by a yearly average of 2.67%. Washington (-2.17%), New York (-2.31%), Oregon (-2.33%), and the District of Columbia (-2.35%) showed the smallest annual percent change. Illinois (-3.34%), Mississippi (-3.04%), Idaho (-2.99%), and Michigan (-2.96%) were the states with the greatest annual decrease. The most common procedures included initial treatment of burns (16000), burn dressing and debridement (16020, 16025, 16030), and burn eschar incision (16035). Medicare reimbursement for burn surgery procedures decreased from 2011 to 2022. The geographic variance in reimbursement patterns may incentivize physicians to pursue other surgical specialties or practice in certain areas which could limit access to care in low reimbursement areas. Further research is needed to examine disparities that may have arisen due to decreasing reimbursement over the last decade. New action is also needed to moderate diminishing burn surgery reimbursement to ensure quality care for Medicare beneficiaries in low-reimbursement states.

## Introduction

In 2023, Medicare was the largest insurer in the United States with a current market share of 18% of the population [1]. Medicare was originally established to provide healthcare for the elderly population with qualifying health conditions. Due to the aging population and increasing Medicare enrolment, it is imperative to examine the impact of Medicare reimbursement for burn surgery procedures on both patients and medical providers [1-3].

Burn surgery physician reimbursement is based on the procedure performed and is codified and billed as a Current Procedural Terminology (CPT) code. The clinic or hospital will utilize the CPT code specific to the procedure or visit to bill the health insurance company for the provided services. CPT codes are assigned a relative value unit (RVU) that determines the physician payout for the procedure performed. Medicare determines total reimbursement by factoring in the work RVUs, practice expense RVUs, and practice liability expense or malpractice RVUs. These values combine to determine the total payout for each CPT code billed for Medicare. The majority of health insurance companies base their reimbursement on Medicare reimbursement rates; therefore, Medicare reimbursement rates will largely influence the total reimbursement received for burn surgery physicians [2,4].

The geographic practice cost index (GPCI) is also utilized to adjust Medicare RVUs. The GPCI varied according to state or territory and year within the study window for this project [2,5]. The GPCI accounts for geographic differences in labor markets, real estate, malpractice insurance, services, supplies, and equipment and attempts to make Medicare physician reimbursement equal across the United States. Due to the GPCI adjustments areas with high desirability and low cost of living will have a negative impact on RVU adjustments. Though the goal of the GPCI is to equalize reimbursement reports indicate GPCI adjustments may not sufficiently assist physicians in rural localities. Despite the impact of the GPCI on geographic differences the agranular representation of GPCI changes will be represented using total national Medicare reimbursement for a given procedure [5].

Since 2000, many surgical specialties have seen decreases in Medicare reimbursement rates. These specialties include neurosurgery, orthopedic surgery, cardiothoracic surgery, and dermatology among other fields [3,6-8]. The changes in reimbursement have many causes primarily among which are congressional budget cuts, Medicare being a balanced budget item, and the role of inflation. Though several studies have evaluated the change in reimbursement rates in various specialties this study is the first to examine regional variation in Medicare reimbursement for burn surgery procedures.

This study identifies regional variation in Medicare reimbursement for burn surgery procedures between 2011 and 2022 in order to highlight financial incentives for burn surgeons to practice in certain localities.

## Materials and methods

This observational analysis utilized the open access data through the Center for Medicare and Medicaid Services Physician Fee Schedule. Due to the nature of open-access data, this project was exempt from the institutional IRB. The University Coding office was consulted to identify the burn surgery-specific CPT codes, which were then confirmed with institutional burn surgeons and the American Burn Association. The code ranges identified for burn surgery procedures were 14000-14302, 15002-15005, 15100-15121, 15271-15278, and 16000-16035 [9]. CPT codes that did not exist for the entirety of the analysis were excluded from the data set.

The Center for Medicare and Medicaid Services Physician Fee Schedule look-up tool was utilized to access reimbursement data from 2011 to 2022. The codes were sorted according to the gross revenue and then utilized for our analysis. Via Mathematica, we identified both the annual and total percent change in revenue for each individual CPT code in each state across the study period. These data were then tabulated according to Medicare Administrative Contractor localities to identify the change in reimbursement for each state and territory from 2011 to 2012.

The Consumer Price Index provided by the United States Department of Labor was utilized to identify yearly inflation rates within the study period. Reimbursement rates for each CPT code were adjusted for inflation in order to give a true inflation-adjusted change in Medicare reimbursement for each state and territory. These calculations were performed using Mathematica and consisted of subtracting the annual inflation rate from the change in Medicare reimbursement for each given year. 

## Results

The average national change in inflation-adjusted Medicare reimbursement for burn surgery procedures from 2011 to 2022 was a decrease of 2.67% on an annual basis. The total change in inflation-adjusted Medicare reimbursement across the 12-year study period was -32.04%. There was geographic variation in the degree of decrease in reimbursement between states. The distribution of national yearly average percentage change in reimbursement was largely regular for the data set.

The range for the national inflation-adjusted yearly percent change was -3.34% in Illinois to -2.17% in Washington state. The mean of the data was -2.67% while the median was -2.73%, indicating a slight positive skew to the data. The highest grossing procedure for burn surgery between 2011 and 2022 was dressing and debridement of a wound, likely due to the high procedure volume when compared to other CPT codes.

**Figure 1:**
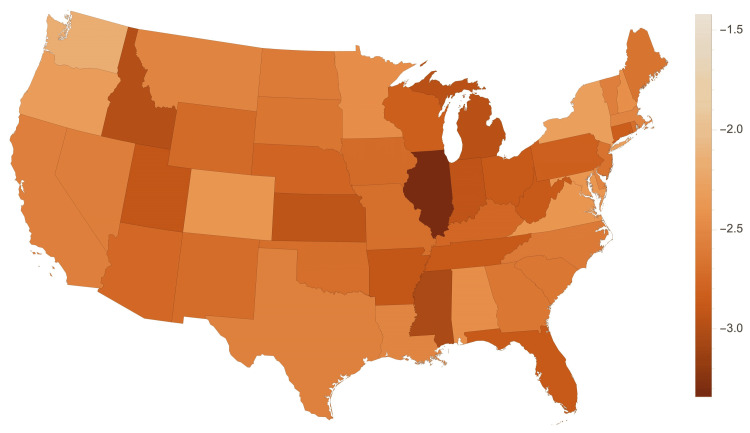
Average annual percent change in Medicare reimbursement by state. The shade of each state corresponds to the percent change labeled in the key. Image credits: Jack Allen and Carson Bateman

Table 1, Table 2, and Figure 1 indicate geographic variance in Medicare reimbursement. Illinois (-3.34%), Mississippi (-3.04%), and Idaho (-2.99%) experienced the sharpest declines in reimbursement. Oregon (-2.33%), New York (-2.31%), and Washington (-2.17%) experienced the lowest decrease in reimbursement rates. Figure 1 is a visual representation of the United States with regions shaded according to the change in Medicare reimbursement. Table 2 present in Appendices provides a written list of the annual change in Medicare reimbursement by state. There was no observed geographic or political pattern in the differences in Medicare reimbursement rates.

**Table 1:** States with both the largest decrease and smallest decrease in annual Medicare reimbursement across the study period.

State or Territory	National Yearly Average Percent Change in Medicare Reimbursement
Illinois	-3.34%
Mississippi	-3.04%
Idaho	-2.99%
Michigan	-2.96%
District of Columbia	-2.35%
Oregon	-2.33%
New York	-2.31%
Washington	-2.17%

## Discussion

This analysis indicates that inflation-adjusted Medicare reimbursement for burn surgery procedures decreased by 2.67% annually over the study period at a national level. National Medicare reimbursement fell by 32.04% over the study period. There were also geographic differences in Medicare reimbursement. This demonstrates a concerning trend for burn surgeons nationally. Though the exact proportion of burn procedures covered by Medicare remains unknown, the diminishing reimbursement will have a financial impact on burn surgeons on a national level. 

Medicare reimbursement rates also have a substantial impact on private health insurance companies’ reimbursement rates [10,11]. Decreasing Medicare reimbursement rates will likely result in an associated decrease in private insurance reimbursement rates, as private insurances use Medicare reimbursement as a benchmark for their reimbursement [11].

The geographic variance in Medicare reimbursement, and therefore in private insurance reimbursement [11], was shown to have variation between states. This trend could provide a financial incentive for burn surgeons to practice in the higher reimbursing areas as opposed to the lower reimbursing areas. There is already a shortage of burn surgeons and burn centers within the United States, and decreasing reimbursement could exacerbate this issue [12]. This study could provide a basis for physicians and hospitals to lobby for increased reimbursement for burn surgery procedures in order to ensure access to quality burn centers across the United States. Other studies indicate existing low access to care for Medicare and Medicaid dual-enrolled beneficiaries [13,14]. Current reimbursement trends may exacerbate this situation and result in further decreases in access to care, especially for dual-enrolled beneficiaries. Further action may be needed to assist burn victims in states with low access to care.

Other specialties also observed decreases in inflation-adjusted Medicare reimbursement over the study period. Dermatology procedures had a 4.8% decrease in reimbursement from 2007-2021 [15]. Orthopedic trauma surgery had a 42% decrease in reimbursement from 2000 to 2020. In addition, emergency medicine had a 29.1% decrease in reimbursement from 2000 to 2020 [16]. The total decrease in inflation-adjusted Medicare reimbursement for burn surgery from 2011 to 2022 was 32%; this is similar to emergency medicine and less than orthopedic trauma surgery, which was among the greatest decreases in reimbursement.

The primary limitation of this study is a lack of access to third-party health insurance company datasets. Though private insurance rates tend to follow Medicare reimbursement rates, we cannot know for certain the exact reimbursement rates of the private sector. Further research is needed in order to examine the impact of decreasing Medicare reimbursement for burn surgery procedures on patient outcomes in the high- and low-reimbursement states.

## Conclusions

Medicare reimbursement for burn surgery procedures decreased from 2011 to 2022 with geographic variation. The geographic variance in reimbursement patterns may incentivize physicians to pursue other surgical specialties or practice in certain areas. This could limit patient access to care, particularly in low-reimbursement areas. New action is needed to moderate diminishing burn surgery reimbursement to ensure quality care for Medicare beneficiaries in low-reimbursement states.

## Appendices

**Table 2:** National yearly inflation-adjusted change in reimbursement values for all United States territories and states.

State or Territory	National Yearly Average Inflation Adjusted Percent Change in Reimbursement	Total Percent Change in Reimbursement
Illinois	-3.336838074	-31.15522759
Mississippi	-3.037965055	-28.77721755
Idaho	-2.986545268	-28.36064368
Michigan	-2.95947158	-28.14041905
Kansas	-2.938522023	-27.96958758
Indiana	-2.930060719	-27.90048595
Utah	-2.914839148	-27.77602294
Arkansas	-2.905064885	-27.69599827
Florida	-2.878691555	-27.47966998
Ohio	-2.874717564	-27.4470222
West Virginia	-2.872901602	-27.43209897
Tennessee	-2.870875455	-27.41544518
Connecticut	-2.835834609	-27.12687892
Wisconsin	-2.827474433	-27.05787779
Pennsylvania	-2.819042415	-26.98822355
Nebraska	-2.781705337	-26.67906648
Kentucky	-2.763105879	-26.52461629
Arizona	-2.755488663	-26.46127749
Wyoming	-2.734893586	-26.28977634
Iowa	-2.727824807	-26.23082881
New Mexico	-2.716706203	-26.13802251
Guam	-2.715076539	-26.12441089
Hawaii	-2.715076539	-26.12441089
Rhode Island	-2.708387796	-26.06851989
Oklahoma	-2.707456279	-26.06073311
Missouri	-2.695069514	-25.9571181
New Jersey	-2.66653826	-25.71795245
Maine	-2.660594394	-25.66803918
South Dakota	-2.646447855	-25.54912179
South Carolina	-2.640335983	-25.49769122
Georgia	-2.637132159	-25.47071858
North Carolina	-2.61240054	-25.26220686
North Dakota	-2.606619063	-25.21338695
Alaska	-2.594133671	-25.10785895
Nevada	-2.578391541	-24.9746117
California	-2.563252125	-24.84626277
Vermont	-2.560470718	-24.82266086
Texas	-2.557415579	-24.79672842
United States Virgin Islands	-2.552075115	-24.75137828
Montana	-2.527422425	-24.54171017
Louisiana	-2.519191136	-24.47158589
Massachusetts	-2.517527577	-24.45740645
Delaware	-2.515094658	-24.43666497
Minnesota	-2.449981835	-23.87962824
Alabama	-2.444587826	-23.83331577
New Hampshire	-2.433317018	-23.73646295
Maryland	-2.390854102	-23.3705623
Colorado	-2.386686241	-23.33456219
Virginia	-2.384619758	-23.31670714
District of Columbia	-2.346943997	-22.9905135
Oregon	-2.328307384	-22.82869339
New York	-2.308445532	-22.6558944
Washington	-2.172125172	-21.4603772
Puerto Rico	-1.422142443	-14.57733292
